# Association of pre-operative estimated GFR on post-operative pulmonary complications in laparoscopic surgeries

**DOI:** 10.1038/s41598-017-06842-4

**Published:** 2017-07-26

**Authors:** Akihiro Shimomura, Yoshitsugu Obi, Reza Fazl Alizadeh, Shiri Li, Ninh T. Nguyen, Michael J. Stamos, Kamyar Kalantar-Zadeh, Hirohito Ichii

**Affiliations:** 1 Department of Surgery, University of California, Irvine, Orange, California USA; 2 Harold Simmons Center for Kidney Disease Research and Epidemiology, Division of Nephrology & Hypertension, University of California, Irvine, Orange, California USA; 3 Fielding School of Public Health, University of California, Los Angeles, Los Angeles, California USA; 4 Los Angeles Biomedical Research Institute, Harbor-University of California, Los Angeles, Torrance, California USA

## Abstract

Despite a large body of evidence showing the pandemic of chronic kidney disease, the impact of pre-operative kidney function on the risk of post-operative pulmonary complications (PPCs) is not well known. We used multivariable logistic regression analyses with 3-level hierarchical adjustments to identify the association of pre-operative estimated glomerular filtration rate (eGFR) with PPCs in laparoscopic surgeries. Among 452,213 patients between 2005 and 2013 in the American College of Surgeons National Surgical Quality Improvement Program (ACS-NSQIP) Database, a total of 3,727 patients (0.9%) experienced PPCs. We found a gradient association between lower eGFR and higher likelihood of PPCs in the unadjusted model. In the case-mix adjusted model, a reverse-J-shaped association was observed; a small albeit significant association with the highest eGFR category emerged. Further adjustment slightly attenuated these associations, but the PPCs risk in the eGFR groups of <30, 30–60, and ≥120 mL/min/1.73 m^2^ remained significant: odds ratios (95% confidence intervals) of 1.82 (1.54–2.16), 1.38 (1.24–1.54), and 1.28 (1.07–1.53), respectively (reference: 90–120 mL/min/1.73 m^2^). Our findings propose a need for careful pre-operative evaluation of cardiovascular and pulmonary functions and post-operative fluid management among patients with not only lower but also very high eGFR.

## Introduction

Post-operative pulmonary complications (PPCs) such as pneumonia, failure to wean off the ventilator, and post-extubation respiratory failure are the most frequent morbidity and causes of death after surgery^1^. The overall incidence of PPCs has been estimated to range from 2.0 to 10.0%^2–4^. The American College of Surgeons National Surgical Quality Improvement Program (ACS-NSQIP), which is a large national cohort, found that PPCs were by far most costly among post-operative complications^5^. Therefore, it may improve patient outcome and reduce medical cost if an efficient strategy is developed to identify and manage patients at risk for PPCs.

Chronic kidney disease (CKD), characterized by decreased kidney function, is a major national and international public health issue with an estimated prevalence of 13.6% in the United States^6^. Additionally, patients with decreased kidney function have higher prevalence of comorbid conditions (e.g., diabetes and cardiovascular disease) and higher mortality than individuals with normal kidney function, and may be more susceptible to PPCs due to diminished ability to maintain fluid balance. Nevertheless, there are scarce data on the relationship between pre-operative kidney function and PPCs.

Laparoscopy has been employed in many surgical procedures to reduce post-operative complications and promote patient recovery^7^. However, the use of laparoscopy can impair respiratory function through the formation of atelectasis and the ventilation-perfusion mismatch caused by the combined effects of supine position and muscle paralysis^8, 9^. Pneumoperitoneum with carbon dioxide during laparoscopic surgeries causes cephalad displacement of the diaphragm and accelerates atelectasis formation^10^, which decreases respiratory compliance and arterial oxygenation^11, 12^. These unfavorable conditions may be aggravated in the presence of CKD.

Based on the above, we examined our hypothesis that patients with pre-operative kidney function would have higher incidence of PPCs after laparoscopic surgery by using the ACS-NSQIP database. We also evaluated the association with non-pulmonary infectious complications in order to contrast the relative contribution of kidney function to different subtypes of post-operative complications.

## Results

### Patient demographic, clinical and laboratory characteristics

**Figure 1:**
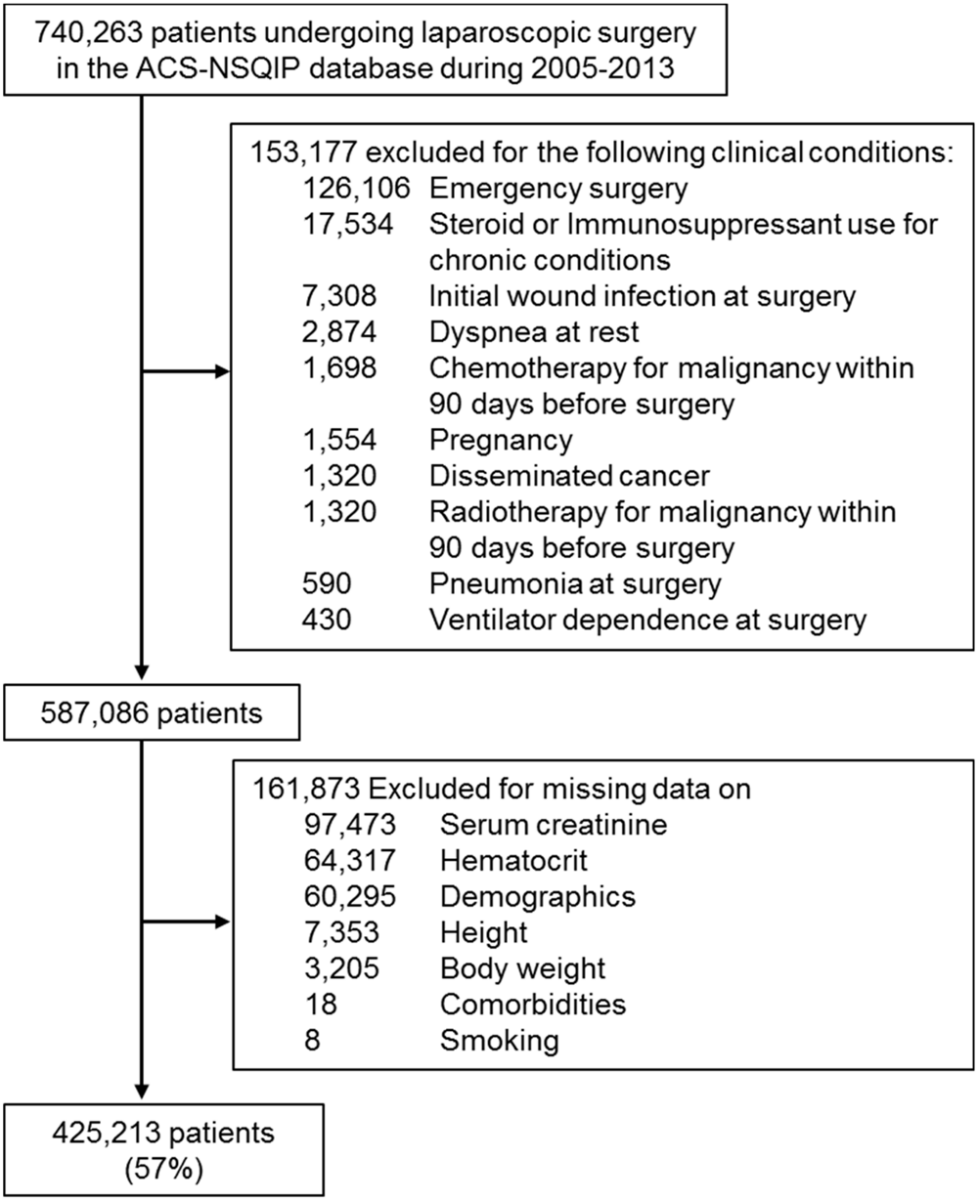
Study flow diagram. Abbreviations: ACS-NSQIP, American College of Surgeons National Surgical Quality Improvement Program.

Out of 740,263 patients undergoing laparoscopic surgery identified in the ACS-NSQIP database from 2005 to 2013, 425,213 patients met our eligibility criteria (Fig. 1). The mean ± SD age of the cohort was 51 ± 16 years old, which included 36% male, 11% black, and 17% smokers (Table 1). Body mass index (BMI) was 33.4 ± 9.6 kg/m^2^. The prevalence of estimated glomerular filtration rate (eGFR) < 30, 30 to <60, 60 to <90, 90 to <120, and ≥120 mL/min/1.73 m^2^ were 1.3% (n = 5,381), 9.1% (n = 38,791), 38.2% (n = 162,516), 43.1% (n = 183,376), and 8.3% (n = 35,149), respectively. Significant trends were observed in all observed variables (*P*_trend_ < 0.001) due to the large sample size. Patients with lower eGFR were older and tended to have a higher prevalence of diabetes, hypertension, chronic obstructive pulmonary disease, dyspnea at moderate exertion, and chronic heart failure, lower concentrations of serum albumin and hematocrit, and a lower prevalence of smoking history.

**Table 1:** Characteristics of patients.

	Missing	Total	eGFR < 30	30 ≤ eGFR < 60	60 ≤ eGFR < 90	90 ≤ eGFR < 120	eGFR ≥ 120
Number of patients		425,213	5,381	38,791	162,516	183,376	35,149
*Surgical procedure*	0%						
Bariatric surgery		23.2%	13.8%	14.8%	19.5%	27.3%	29.2%
Cholecystectomy		28.7%	34.4%	28.4%	26.3%	28.5%	39.9%
Others		48.2%	51.8%	56.8%	54.2%	44.3%	30.9%
BMI (kg/m^2^)	0%	33.4 ± 9.6	32.1 ± 9.0	32.2 ± 8.8	32.7 ± 8.9	34.3 ± 10.0	35.2 ± 11.2
Age (yr)	0%	51 ± 16	63 ± 15	69 ± 12	58 ± 14	46 ± 12	31 ± 9
Men	0%	35.5%	47.2%	39.5%	40.3%	32.8%	21.6%
*Race*	0%						
Black		10.5%	19.5%	6.8%	7.0%	10.1%	32.3%
White		83.8%	72.3%	88.3%	88.3%	83.7%	60.2%
Diabetes	0%	15.6%	41.1%	28.4%	15.2%	13.6%	9.2%
Hypertension	0%	44.3%	83.6%	77.9%	51.4%	35.1%	16.9%
History of COPD	0%	2.6%	6.8%	5.9%	3.1%	1.7%	0.4%
Dyspnea at moderate exertion	0%	8.8%	15.4%	14.0%	9.2%	7.7%	5.8%
History of CHF	0%	0.3%	2.8%	1.1%	0.3%	0.1%	0.0%
Smoker	0%	16.5%	12.5%	9.6%	13.4%	19.7%	22.2%
*Laboratories*							
Alb (g/dL)	28.1%	4.0 ± 0.5	3.6 ± 0.7	3.9 ± 0.6	4.0 ± 0.5	4.0 ± 0.5	3.9 ± 0.6
Hct (%)	0%	39.8 ± 4.5	35.2 ± 5.3	38.6 ± 5.0	40.4 ± 4.4	40.0 ± 4.3	38.3 ± 4.5
Estimated GFR (mL/min/1.73 m^2^)	0%	89.3 ± 23.4	15.6 ± 8.8	49.8 ± 7.8	77.2 ± 8.3	103.1 ± 8.2	128.8 ± 8.0

Values are expressed as means ± SD or percentage, appropriately. Abbreviations: BMI, body mass index; COPD, chronic obstructive pulmonary disease; CHF, chronic heart failure; Alb, albumin; Hct, hematocrit; eGFR, estimated glomerular filtration rate.

### Association of estimated GFR with post-operative pulmonary complications

A total of 3,727 patients (0.9%) experienced PPCs (i.e., pneumonia, unplanned intubation, and/or on ventilator for over 48 hours) within 30 days after surgery. There was an increasing trend in the rate of PPCs across lower eGFR categories; the incidence of PPCs was 0.4%, 0.6%, 0.9%, 2.2%, and 3.6% in patients with eGFR ≥ 120, 90 to <120, 60 to <90, 30 to <60, and <30 mL/min/1.73 m^2^, respectively (Table 2).

**Table 2:** Details of pulmonary complications.

	Total	eGFR < 30	30 ≤ eGFR < 60	60 ≤ eGFR < 90	90 ≤ eGFR < 120	eGFR ≥ 120	*P*. value
Number of patients	425,213	5,381	38,791	162,516	183,376	35,149	
*Respiratory complications*	3,727 (0.9%)	192 (3.6%)	853 (2.2%)	1,482 (0.9%)	1,050 (0.6%)	150 (0.4%)	<0.001
Pneumonia	2,146 (0.5%)	83 (1.5%)	431 (1.1%)	853 (0.5%)	674 (0.4%)	105 (0.3%)	<0.001
Ventilator >48 hours	1,197 (0.3%)	70 (1.3%)	294 (0.8%)	467 (0.3%)	327 (0.2%)	39 (0.1%)	<0.001
Unplanned re-intubation	1,734 (0.4%)	109 (2.0%)	471 (1.2%)	672 (0.4%)	438 (0.2%)	44 (0.1%)	<0.001

**Figure 2:**
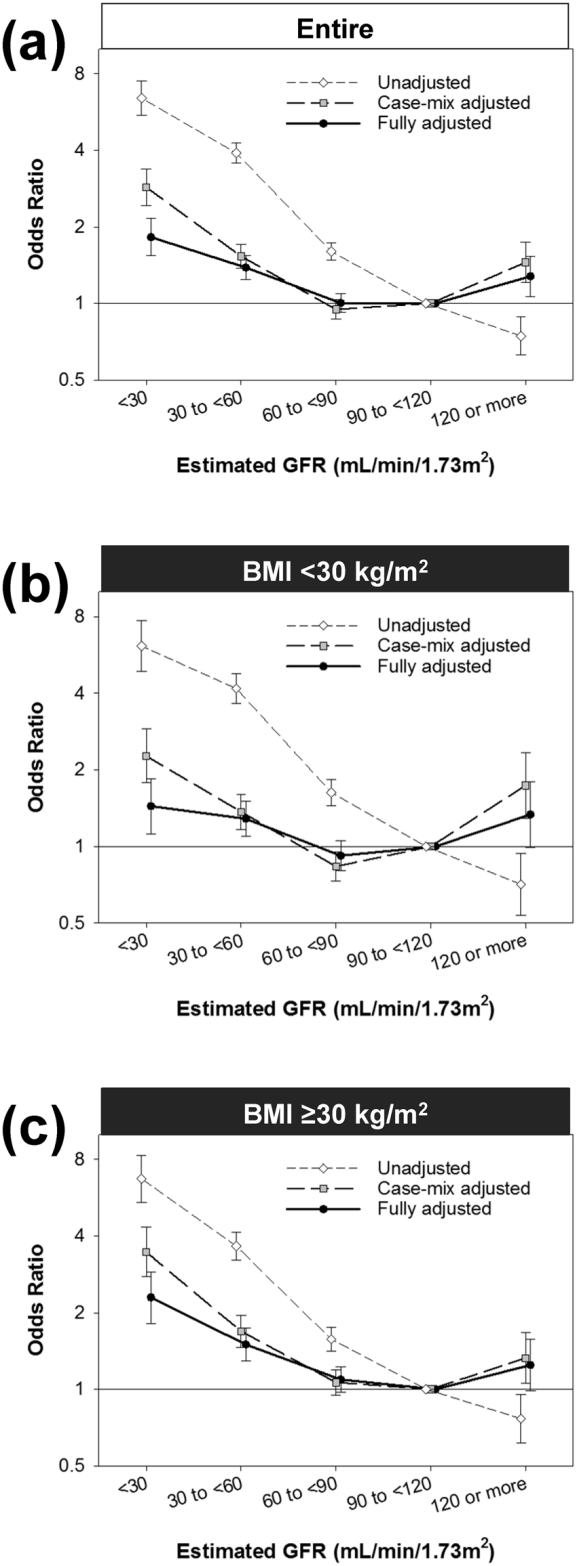
Estimated GFR and risk of post-operative pulmonary complications. Multivariable logistic regression analyses were conducted to examine the association of estimated GFR with post-operative pulmonary complications among 425,213 patients who underwent laparoscopic surgery (2005–2013) with three levels of adjustment stratified by body mass index (BMI), (reference: 90 to <120 mL/min/1.73 m^2^): (**a**) Entire, (**b**) BMI < 30 kg/m^2^, (**c**) BMI ≥ 30 kg/m^2^. Points and lines represent the point estimates and 95% CIs, respectively. Abbreviations: GFR, glomerular filtration rate.

Lower eGFR categories showed an incremental association with higher likelihood of PPCs in the unadjusted model; <30, 30 to <60, 60 to <90, and ≥120 mL/min/1.73 m^2^ in eGFR showed odds ratios (ORs) (95% confidence intervals [CIs]) of 6.42 (5.50 to 7.51), 3.90 (3.56 to 4.28), 1.60 (1.48 to 1.73), and 0.74 (0.63 to 0.88), respectively, (reference: 90 to <120 mL/min/1.73 m^2^; *P*_trend_ < 0.001; Fig. 2). In the case-mix adjusted model, there appeared a reverse J-shape association; the likelihood of PPCs in the lower eGFR categories (i.e., <90 mL/min/1.73 m^2^) were attenuated while the PPCs risk associated with the highest eGFR category (i.e., ≥120 mL/min/1.73 m^2^) reversed and turned significantly high (OR 1.45 [95% CI, 1.21–1.74]). The fully adjusted model showed slightly attenuated associations, but the PPCs risk in the eGFR groups of <30, 30 to 60, and >120 mL/min/1.73 m^2^ retained statistical significance; <30, 30 to <60, and ≥120 mL/min/1.73 m^2^ in eGFR showed ORs (95% CIs) of 1.82 (1.54 to 2.16), 1.38 (1.24 to 1.54), and 1.28 (1.07 to 1.53), respectively (Supplemental Table S1). Consistent findings were observed in the restricted spline models by using eGFR as a continuous variable (Supplement Figure S1). Area under the receiver operating characteristic curve (AUROC) were 0.63, 0.71, and 0.76 in the unadjusted, case-mix adjusted, and fully adjusted model, respectively.

**Figure 3:**
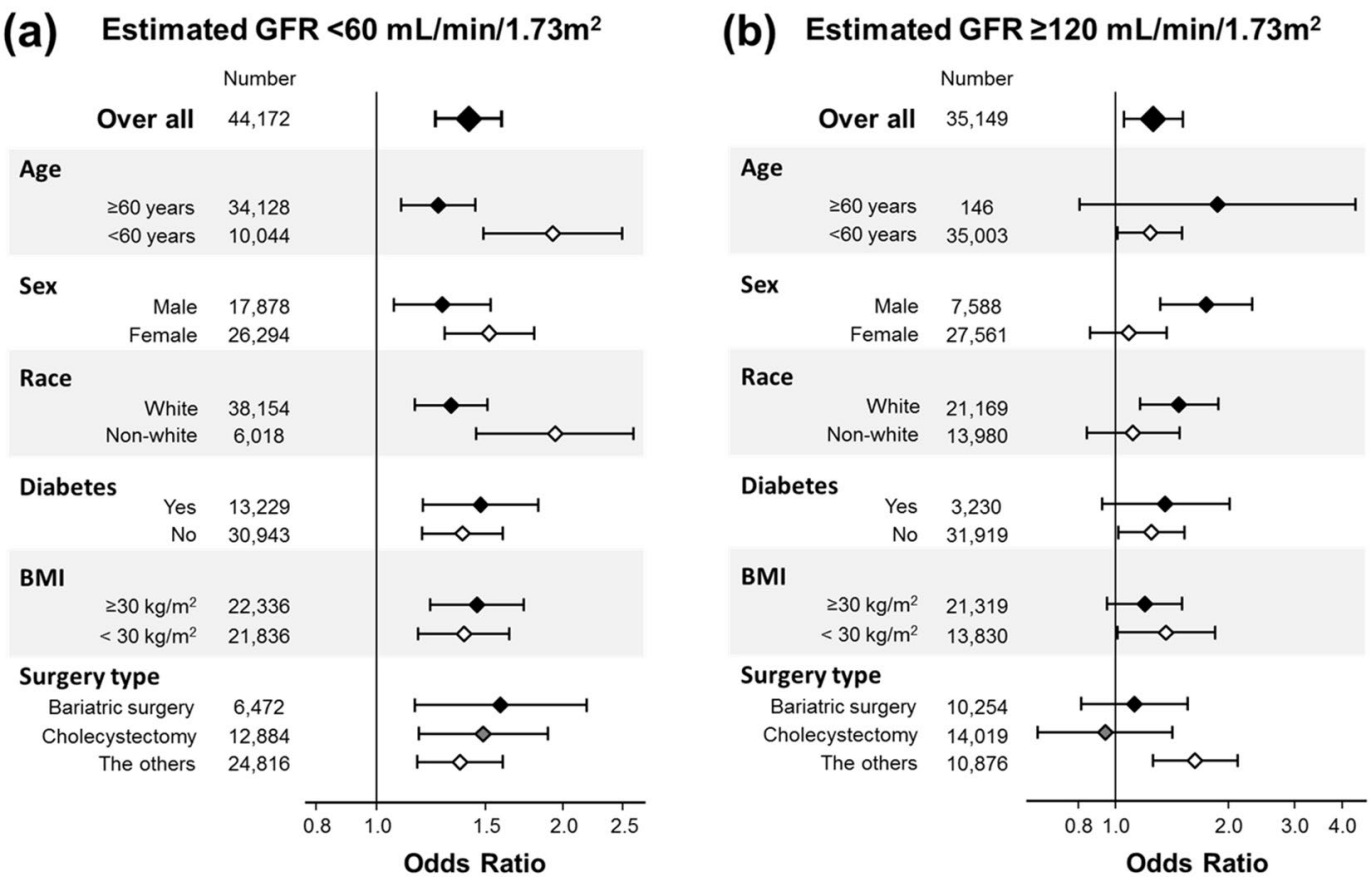
Overall and subgroup analyses of associations between eGFR and risk of post-operative pulmonary complications. Overall and subgroup analyses of associations between eGFR and risk of post-operative pulmonary complications among 425,213 patients who underwent laparoscopic surgery (2005–2013) in the fully adjusted model. Points and bars represent OR estimates and 95% CIs, respectively, (reference: 60 to <120 mL/min/1.73 m^2^): (**a**) eGFR < 60 mL/min/1.73 m^2^, (**b**) eGFR ≥ 120 mL/min/1.73 m^2^. Abbreviations: GFR, glomerular filtration rate; BMI, body mass index.

Then we examined if obese status modifies the association between eGFR and PPCs. The interaction term between BMI and eGFR was significant in the unadjusted model (*P*_interaction_ = 0.03). However, it lost statistical significance with further adjustment (*P*_interaction_ = 0.06 and 0.31 in the case-mix adjusted and fully-adjusted model, respectively) and the eGFR-PPCs association was consistent between obese vs. non-obese patients (i.e., BMI ≥ 30 vs. <30 kg/m^2^) (Fig. 2B and C). The adjusted association between low eGFR (i.e., <60 mL/min/1.73 m^2^) and PPCs was robust and strong in subgroup analyses based on sex (male vs. female), race (white vs. non-white), diabetes (with vs. without), BMI (≥30 vs. <30 kg/m^2^), and surgery type (bariatric surgery, cholecystectomy, vs. the others) and the PPCs risk associated with low eGFR was especially pronounced in younger patients (i.e., <60 years old; *P*_interaction_ = 0.04) (Fig. 3A).The adjusted association between high eGFR (i.e., ≥120 mL/min/1.73 m^2^) and PPCs was small and inconsistent for sex; high eGFR was associated with higher risk of PPCs among male patients but not among female patients (*P*_interaction_ < 0.001) (Fig. 3B).

### Association of estimated GFR with post-operative non-pulmonary infectious complications

A total of 16,428 patients (3.9%) experienced post-operative non-pulmonary infectious complications (i.e., sepsis, septic shock, urinary tract infection, superficial incisional surgical site infection [SSI], deep incisional SSI and/or organ or space SSI) within 30 days after surgery. There was an increasing trend in the rate of post-operative non-pulmonary infectious complications across lower eGFR categories; the incidence of PPCs was 2.9%, 3.6%, 4.0%, 5.2%, and 7.0% in patients with eGFR ≥ 120, 90 to <120, 60 to <90, 30 to <60, and <30 mL/min/1.73 m^2^, respectively (Table 3).

**Table 3:** Association of estimated GFR with post-operative non-pulmonary infectious complications.

	Total	eGFR < 30	30 ≤ eGFR < 60	60 ≤ eGFR < 90	90 ≤ eGFR < 120	eGFR ≥ 120	*P*. value
Number of patients	425,213	5,381	38,791	162,516	183,376	35,149	
*Infectious complications*	16,428 (3.9%)	376 (7.0%)	1,997 (5.2%)	6,431 (4.0%)	6,600 (3.6%)	1,024 (2.9%)	<0.001
Sepsis	3,361 (0.8%)	110 (2.0%)	430 (1.1%)	1,275 (0.8%)	1,303 (0.7%)	244 (0.7%)	<0.001
Septic shock	1,106 (0.3%)	83 (1.5%)	248 (0.6%)	432 (0.3%)	310 (0.2%)	33 (0.1%)	<0.001
Urinary tract infection	4,559 (1.1%)	103 (1.9%)	675 (1.7%)	1,815 (1.1%)	1,668 (0.9%)	298 (0.9%)	<0.001
Superficial incisional surgical site infection	6,300 (1.5%)	73 (1.4%)	608 (1.6%)	2,507 (1.5%)	2,748 (1.5%)	364 (1.0%)	<0.001
Deep incisional surgical site infection	915 (0.2%)	20 (0.4%)	102 (0.3%)	344 (0.2%)	391 (0.2%)	58 (0.2%)	0.006
Organ or space surgical site infection	3,595 (0.9%)	71 (1.3%)	364 (0.9%)	1,406 (0.9%)	1,528 (0.8%)	226 (0.6%)	<0.001

**Figure 4:**
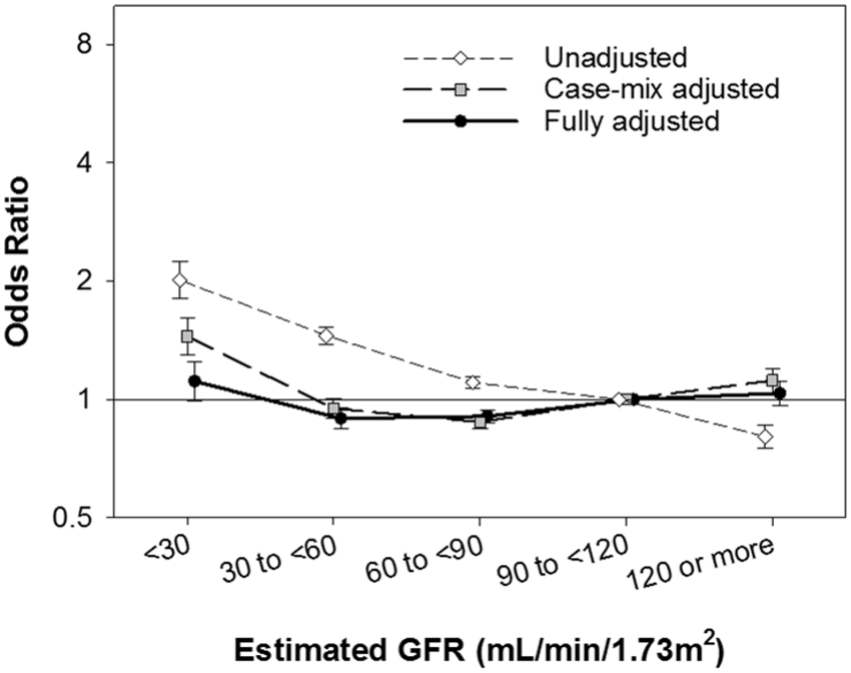
Estimated GFR and risk of post-operative non-pulmonary infectious complications. Multivariable logistic regression analyses were conducted to examine the association of estimated GFR with post-operative non-pulmonary infectious complications among 425,213 patients who underwent laparoscopic surgery (2005–2013) with three levels of adjustment. Points and lines represent the point estimates and 95% CIs, respectively, (reference: 90 to <120 mL/min/1.73 m^2^). Abbreviations: GFR, glomerular filtration rate.

Lower eGFR categories showed a gradient association with higher likelihood of non-pulmonary infectious complications in the unadjusted model as observed for PPCs. (*P*_trend_ < 0.001, Fig. 4). Although there was also a significant trend in the risk of non-pulmonary infectious complications across eGFR categories (*P*_trend_ < 0.001 in all adjustment models), the risk associated with eGFR categories was much smaller compared to PPCs; <30, 30 to <60, 60 to <90, and ≥120 mL/min/1.73 m^2^ in eGFR categories showed fully-adjusted ORs (95% CIs) of 1.11 (0.99 to 1.24), 0.89 (0.84 to 0.95), 0.91 (0.87 to 0.94), and 1.04 (0.96 to 1.11), respectively, (reference: 90 to <120 mL/min/1.73 m^2^) (Supplemental Table S2).

## Discussion

This is the largest study, to our knowledge, which has examined the risk of post-operative complications after abdominal surgeries associated with preoperative eGFR, an index of kidney function, using a large contemporary national database of surgical outcomes with prospectively recorded assessments. Our study is also the first to demonstrate that the adjusted risk of PPCs were higher in the range of not only lower but also higher eGFR levels (i.e., <60 and ≥120 mL/min/1.73 m^2^, respectively). In contrast, there was no apparent association between eGFR and non-pulmonary infectious complications, supporting a close link between kidney function and pulmonary complications.

There are several studies showing the association of kidney dysfunction with post-operative complications, but most of them examined a composite of heterogeneous outcomes^13–15^. Only a few research groups have reported the relationship between kidney dysfunction and higher risk of cause-specific complications including PPCs and non-pulmonary infectious complications^16, 17^, and they focused on cardiac surgeries which account for only <1% of the all surgeries^18^. Our study strengthened the evidence of PPCs risk associated with kidney dysfunction by extending the applicable area to laparoscopic surgeries that are much more frequently conducted (approximately 25% in the ACS-NSQIP database) and generally considered low in morbidity risk. Meanwhile, our study did not find an increased risk of non-pulmonary infectious complications among patients with reduced kidney function, which may reflect the lower baseline risk underlying laparoscopic surgeries vs. cardiac surgeries.

The exact underlying mechanisms of the association between PPCs and lower eGFR remain unclear, but patients with decreased kidney function are less capable to maintain fluid balance by urine in the setting of post-operative fluid infusion, which may lead to hypervolemia and cardiogenic pulmonary edema^19, 20^. Second, elevated fibroblast growth factor 23 (FGF23) levels and vitamin D deficiency may contribute to the development of PPCs. FGF23 increases in the very early course of CKD^21–23^, and vitamin D deficiency is also commonly observed in CKD especially among those with proteinuria and diabetes^24–27^. Several study have reported that both elevated FGF23 and vitamin D deficiency may cause left ventricular hypertrophy via activation of the calcineurin pathway^28–30^ and the renin-angiotensin-aldosterone system^31–33^, respectively. Indeed, patients with CKD are at high risk of cardiovascular events and death even in early stages of CKD^34–36^. Third, patients with CKD may have impaired respiratory muscle strength due to protein-energy wasting^37–40^. Fourth, hypoalbuminemia, which is often observed among patients with CKD, may induce pulmonary congestion. Hypoalbuminemia decrease plasma oncotic pressure and thus promotes movement of fluid out of the pulmonary capilaries^41, 42^. Indeed, the risk of PPCs associated with eGFR was attenuated in the fully-adjusted model including serum albumin, suggesting the link between hypoalbuminemia and protein-energy wasting.

We observed a significant effect modification by age against the association between low GFR and PPCs; the PPCs risk associated with eGFR < 60 mL/min/1.73 m^2^ was pronounced among younger vs. older patients (i.e., <60 vs. ≥60 years old). The exact reason for this finding is unclear, but there may be selection bias where older patients underwent invasive therapeutic procedures (e.g., bariatric surgery) only if they do not have apparent health-related problems.

The association between the highest eGFR group and higher likelihood of PPCs may be explained by frailty and hyperfiltration. Estimated GFR is well known to overestimate actual kidney function in subjects with low muscle mass, and thus frail patients with normal kidney function may have falsely high eGFR and high susceptibility to fluid overload due to weak respiratory muscle. Among female patients, the PPCs risk associated with eGFR ≥ 120 mL/min/1.73 m^2^ was not significant probably because female patients, compared to male patients, are likely to have less muscle mass, which might have attenuated the association between PPCs and high eGFR induced by low serum creatinine concentrations. Meanwhile, glomerular hyperfiltration, often defined as GFR ≥ 120 mL/min/1.73 m^2^ ^43^, is caused by various clinical conditions such as diabetes, obesity, and sleep apnea^44^, and may be a marker of early renal damage in pre-diabetes and pre-hypertension^45^. Second, endothelial dysfunction observed in CKD may increase capillary leak leading to non-cardiogenic pulmonary edema^46^. Of note, endothelial dysfunction is observed even in pre-diabetes and pre-hypertension, which can cause glomerular hypertension resulting in proteinuria and hyperfiltration at the very beginning period of CKD^45, 47, 48^. These findings may explain the positive correlation between high eGFR and pulmonary complication risk even after adjustment for diabetes, hypertension and BMI.

Our study has several limitations. First, as the retrospective nature of the observational study, we are not able to estimate the cause-effect relationships, and there may be residual confounding and/or unmeasured cofounders that might have biased the estimated association. For example, the wide variation in hospital setting, hospital quality, surgical strategy, and surgeons’ expertise may confound our study because the ACS-NSQIP database was collected from about 500 hospitals across the United States. Coding errors could potentially have occurred when data was collected by untrained clinical reviewers^49^. Additionally, the ACS-NSQIP did not record some relevant information such as echocardiography, pulmonary function test, serum levels of FGF23 and 25-hydroxyvitamin D, pleural effusion, obstructive sleep apnea, and asthma. Second, the study population was comprised of patients admitted to hospitals participating in the ACS-NSQIP system and therefore may not reflect all patients across the United States. Third, 97,473 patients (16.6%) were missing serum creatinine values among patients included in this study, and available serum creatinine measures may not be representative of the entire source population. Potential selection bias may exist, such that physicians at participant institutions may be unlikely to re-measure serum creatinine levels in stable patients with serum creatinine data measured by primary care physicians. Fourth, preoperative eGFR may not represent patient kidney function in a stable condition because low eGFR in this study may indicate acute kidney injury in the preoperative period. Exclusion of the emergency cases is our best effort to avoid the influence of acute kidney injury on the association between eGFR and PPCs.

In conclusion, both low and extremely high eGFR levels are associated with higher risk of post-operative pulmonary complications, but not non-pulmonary infectious complications, after laparoscopic surgery. Careful preoperative evaluation of cardiovascular and pulmonary functions and post-operative management may be necessary among patients with abnormal eGFR.

## Methods

### Database

The ACS-NSQIP is a risk adjusted, surgical outcomes-based program designed to measure and improve the quality of surgical care^50–52^. Trained clinical reviewers prospectively collect the ACS-NSQIP data and validated data from medical records on preoperative risk factors, preoperative laboratory values, intraoperative variables, 30-day postoperative mortality, and 30-day morbidity on all patients undergoing major surgeries at participant institutions^50–52^. In 2013, the ACS-NSQIP database contained patient data for more than 2.9 million cases from 435 participant hospitals.

### Patient selection

We used the ACS-NSQIP database (2005 to 2013) to extract all patients who underwent laparoscopic surgeries with the following current procedural terminology (CPT) codes: cholecystectomy (47562–47564), gastroesophageal reflux (43279, 43280, and 43283), paraesophageal hernia (43280–43282), bariatric surgery (43644, 43645, 43659, and 43770–43775), colectomy (44204–44208 and 44210–44212), inguinal hernia (49650 and 49651), incisional hernia (49652–49657), adrenalectomy (60650), appendectomy (44970 and 44979), splenectomy (38120), colostomy (44188, 44206, and 44208), exteriorization of intestine (44227), ileostomy (44186 and 44187), proctectomy (45395, 45397, 45400, 45402, and 45499), nephrectomy (50543, 50545, 50546, and 50548), prostatectomy (55866), hysterectomy (58541–58544, 58548, 58550, 58552–58554, and 58570–58573), and myomectomy (58545 and 58546) (Supplemental Table S3). We excluded 153,177 patients who had a history of chemotherapy, had an initial wound infection at the time of surgery, underwent emergency surgeries, were pregnant, had disseminated cancers, used steroids or immunosuppressants for a chronic condition, were depend on a ventilator at the time of surgery, had pneumonia at the time of surgery, or had dyspnea at rest (Fig. 1). A total of 161,873 patients were also excluded for missing values on serum creatinine (excluding on maintenance dialysis), age, sex, race, height, body weight, relevant comorbid conditions (i.e., hypertension, diabetes, chronic obstructive pulmonary disease, chronic heart failure, and dyspnea), smoking or hematocrit. The final analytic cohort was comprised of 425,213 patients who underwent laparoscopic surgery.

### Study variables

The primary exposure of interest was kidney function expressed as eGFR. Preoperative serum creatinine concentrations, age, sex, and race were used to calculate eGFR based on the Chronic Kidney Disease Epidemiology Collaboration (CKD-EPI) formula^53^. Patients not on maintenance dialysis were then categorized into five group based on eGFR (<30, 30 to <60, 60 to <90, 90 to <120, and 120 mL/min/1.73 m^2^ or more) while patients with end-stage renal disease requiring dialysis were considered to have eGFR <30 mL/min/1.73 m^2^ irrespective of their eGFR values. BMI was also calculated for each patient using height and body weight. The association of these eGFR categories with each patient characteristic was evaluated by non-parametric trend test^54^. The primary outcome was PPCs defined as pneumonia, unplanned intubation, or on ventilator for over 48 hours within 30 days after surgery. Comorbid factors were obtained in preoperative period.

### Statistical analyses

Patient characteristics are expressed as means ± SD, or percentages, as appropriate. Differences between included and excluded patients were compared by standardized differences due to the large sample size of this study^55, 56^.

Missing covariate data of serum concentrations of albumin was imputed using multiple imputation (MI) on the basis of the variables of age, sex, race, smoking, hematocrit, eGFR, and four comorbid conditions including chronic obstructive pulmonary disease, hypertension, diabetes, and chronic heart failure. We created five MI datasets using the multivariable normal model. In the MI analyses, the analysis of each dataset was carried out separately, and then five sets of estimates (odds ratios) are combined to generate a single set of estimates^57^.

Multivariable logistic regression analyses were conducted to examine the association of kidney function with PPCs or non-pulmonary infectious complications using eGFR categories. Estimated GFR was also modeled as continuous variables, where patients with end-stage renal disease requiring dialysis were considered to have eGFR of 7.5 mL/min/1.73 m^2^ (i.e., a half value of the upper limit GFR of stage 5 CKD), and their relationship with the outcome was examined using restricted cubic spline functions with a reference at 90 mL/min/1.73 m^2^ and four knots at the 5th, 35th, 65th, 95th percentile of eGFR. Three levels of hierarchical adjustments were used as follows: (1) unadjusted models; (2) case-mix adjusted models that included age, sex, race and BMI; (3) fully adjusted models that included the above plus smoking, hematocrit, serum concentrations of albumin, and four comorbid conditions described above. Effect modifications against the association of eGFR with PPCs by age (≥60 or <60 years old), sex, race (white or non-white), diabetes, BMI (≥30 or <30 kg/m^2^), and surgery type (bariatric surgery, cholecystectomy, or the others) were examined by including each interaction term into the fully adjusted model.

All analyses were carried out with STATA MP version 13.1 (StataCorp, College Station, TX), and *P* values less than 0.05 were considered statistical significance.

## Electronic supplementary material

Supplemental Materials

## References

[1] Canet J, Mazo V (2010). Postoperative pulmonary complications. Minerva Anestesiol.

[2] Arozullah AM, Daley J, Henderson WG, Khuri SF (2000). Multifactorial risk index for predicting postoperative respiratory failure in men after major noncardiac surgery. The National Veterans Administration Surgical Quality Improvement Program. Ann Surg.

[3] Khuri, S. F. *et al*. Determinants of long-term survival after major surgery and the adverse effect of postoperative complications. *Ann Surg* **242**, 326–341, discussion 341–323 (2005). 10.1097/01.sla.0000179621.33268.83 PMC1357741 16135919

[4] Johnson RG (2007). Multivariable predictors of postoperative respiratory failure after general and vascular surgery: results from the patient safety in surgery study. J Am Coll Surg.

[5] Dimick JB (2004). Hospital costs associated with surgical complications: a report from the private-sector National Surgical Quality Improvement Program. J Am Coll Surg.

[6] Saran, R. et al. US Renal Data System 2014 Annual Data Report: Epidemiology of Kidney Disease in the United States. *Am J Kidney Dis* **66**, Svii, S1–305, doi:10.1053/j.ajkd.2015.05.001 (2015). 10.1053/j.ajkd.2015.05.001 PMC6643986 26111994

[7] Rockall TA, Demartines N (2014). Laparoscopy in the era of enhanced recovery. Best Pract Res Clin Gastroenterol.

[8] Hedenstierna G, Edmark L (2005). The effects of anesthesia and muscle paralysis on the respiratory system. Intensive Care Med.

[9] Hedenstierna G, Rothen HU (2000). Atelectasis formation during anesthesia: causes and measures to prevent it. J Clin Monit Comput.

[10] Meininger D, Byhahn C, Mierdl S, Westphal K, Zwissler B (2005). Positive end-expiratory pressure improves arterial oxygenation during prolonged pneumoperitoneum. Acta Anaesthesiol Scand.

[11] Hazebroek EJ, Haitsma JJ, Lachmann B, Bonjer HJ (2002). Mechanical ventilation with positive end-expiratory pressure preserves arterial oxygenation during prolonged pneumoperitoneum. Surg Endosc.

[12] Takahata O (2007). Effect of age on pulmonary gas exchange during laparoscopy in the Trendelenburg lithotomy position. Acta Anaesthesiol Scand.

[13] Turgeon NA (2012). The impact of renal function on outcomes of bariatric surgery. J Am Soc Nephrol.

[14] Saleh F, Kim SJ, Okrainec A, Jackson TD (2015). Bariatric surgery in patients with reduced kidney function: an analysis of short-term outcomes. Surg Obes Relat Dis.

[15] Ekici Y, Tezcaner T, Aydoğan C, Karakayalı FY, Moray G (2014). Outcomes of Laparoscopic Cholecystectomy in Patients with End-Stage Renal Disease. International Journal of Clinical Medicine.

[16] Cooper WA (2006). Impact of renal dysfunction on outcomes of coronary artery bypass surgery: results from the Society of Thoracic Surgeons National Adult Cardiac Database. Circulation.

[17] Zakeri R (2005). Relation between mild renal dysfunction and outcomes after coronary artery bypass grafting. Circulation.

[18] Hall MJ, DeFrances CJ, Williams SN, Golosinskiy A, Schwartzman A (2010). National Hospital Discharge Survey: 2007 summary. Natl Health Stat Report.

[19] Grassi V, Malerba M, Boni E, Tantucci C, Sorbini CA (1994). Uremic lung. Contrib Nephrol.

[20] Obi Y, Kim T, Kovesdy CP, Amin AN, Kalantar-Zadeh K (2015). Current and Potential Therapeutic Strategies for Hemodynamic Cardiorenal Syndrome. Cardiorenal Medicine.

[21] Nakano C (2012). Combined use of vitamin D status and FGF23 for risk stratification of renal outcome. Clin J Am Soc Nephrol.

[22] Al-Badr W, Martin KJ (2008). Vitamin D and kidney disease. Clin J Am Soc Nephrol.

[23] Pereira RC (2009). Patterns of FGF-23, DMP1, and MEPE expression in patients with chronic kidney disease. Bone.

[24] Hamano T (2011). Guideline-practice gap in the management of predialysis chronic kidney disease mineral bone disorder in Japan. Ther Apher Dial.

[25] Tanaka H (2009). The impact of diabetes mellitus on vitamin D metabolism in predialysis patients. Bone.

[26] Obi Y (2014). Vitamin D deficiency predicts decline in kidney allograft function: a prospective cohort study. J Clin Endocrinol Metab.

[27] Obi Y, Hamano T, Isaka Y (2015). Prevalence and prognostic implications of vitamin D deficiency in chronic kidney disease. Dis Markers.

[28] Gutierrez OM (2009). Fibroblast growth factor 23 and left ventricular hypertrophy in chronic kidney disease. Circulation.

[29] Faul C (2011). FGF23 induces left ventricular hypertrophy. J Clin Invest.

[30] Grabner A (2015). Activation of Cardiac Fibroblast Growth Factor Receptor 4 Causes Left Ventricular Hypertrophy. Cell Metab.

[31] Xiang W (2005). Cardiac hypertrophy in vitamin D receptor knockout mice: role of the systemic and cardiac renin-angiotensin systems. Am J Physiol Endocrinol Metab.

[32] Lee JH, O’Keefe JH, Bell D, Hensrud DD, Holick MF (2008). Vitamin D deficiency an important, common, and easily treatable cardiovascular risk factor?. J Am Coll Cardiol.

[33] Judd SE, Tangpricha V (2009). Vitamin D deficiency and risk for cardiovascular disease. Am J Med Sci.

[34] Go AS, Chertow GM, Fan D, McCulloch CE, Hsu CY (2004). Chronic kidney disease and the risks of death, cardiovascular events, and hospitalization. N Engl J Med.

[35] Obi Y (2010). Impact of age and overt proteinuria on outcomes of stage 3 to 5 chronic kidney disease in a referred cohort. Clin J Am Soc Nephrol.

[36] Clase CM (2011). Estimated glomerular filtration rate and albuminuria as predictors of outcomes in patients with high cardiovascular risk: a cohort study. Ann Intern Med.

[37] Bark H, Heimer D, Chaimovitz C, Mostoslovski M (1988). Effect of Chronic Renal-Failure on Respiratory Muscle Strength. Respiration.

[38] Kovelis D (2008). Pulmonary function and respiratory muscle strength in chronic renal failure patients on hemodialysis. J Bras Pneumol.

[39] Teixeira CG, Duarte Mdo C, Prado CM, Albuquerque EC, Andrade LB (2014). Impact of chronic kidney disease on quality of life, lung function, and functional capacity. J Pediatr (Rio J).

[40] Obi Y, Qader H, Kovesdy CP, Kalantar-Zadeh K (2015). Latest consensus and update on protein-energy wasting in chronic kidney disease. Curr Opin Clin Nutr Metab Care.

[41] Pierson DJ (2006). Respiratory considerations in the patient with renal failure. Respir Care.

[42] Turcios NL (2012). Pulmonary complications of renal disorders. Paediatr Respir Rev.

[43] Cachat F, Combescure C, Cauderay M, Girardin E, Chehade H (2015). A systematic review of glomerular hyperfiltration assessment and definition in the medical literature. Clin J Am Soc Nephrol.

[44] Helal I, Fick-Brosnahan GM, Reed-Gitomer B, Schrier RW (2012). Glomerular hyperfiltration: definitions, mechanisms and clinical implications. Nat Rev Nephrol.

[45] Palatini P (2012). Glomerular hyperfiltration: a marker of early renal damage in pre-diabetes and pre-hypertension. Nephrol Dial Transplant.

[46] Gibson DG (1966). Haemodynamic Factors in Development of Acute Pulmonary Oedema in Renal Failure. Lancet.

[47] Yilmaz MI (2006). The determinants of endothelial dysfunction in CKD: oxidative stress and asymmetric dimethylarginine. Am J Kidney Dis.

[48] Malyszko J (2010). Mechanism of endothelial dysfunction in chronic kidney disease. Clin Chim Acta.

[49] Lorence DP, Ibrahim IA (2003). Benchmarking variation in coding accuracy across the United States. J Health Care Finance.

[50] Khuri SF (1998). The Department of Veterans Affairs’ NSQIP: the first national, validated, outcome-based, risk-adjusted, and peer-controlled program for the measurement and enhancement of the quality of surgical care. National VA Surgical Quality Improvement Program. Ann Surg.

[51] Fink, A. S. *et al*. The National Surgical Quality Improvement Program in non-veterans administration hospitals: initial demonstration of feasibility. *Ann Surg* **236**, 344–353; discussion 353–344, doi:10.1097/01.SLA.0000027082.79556.55 (2002). 10.1097/00000658-200209000-00011 PMC1422588 12192321

[52] Khuri SF (2005). The NSQIP: a new frontier in surgery. Surgery.

[53] Levey AS (2009). A new equation to estimate glomerular filtration rate. Ann Intern Med.

[54] KDIGO 2012 Clinical Practice Guideline for the Evaluation and Management of Chronic Kidney Disease. *Kidney international supplements* **3**, 1–150, doi:10.1038/kisup.2012.72 (2013). 10.1038/ki.2013.243 23989362

[55] Austin PC (2009). Balance diagnostics for comparing the distribution of baseline covariates between treatment groups in propensity-score matched samples. Stat Med.

[56] Schacht A, Bogaerts K, Bluhmki E, Lesaffre E (2008). A new nonparametric approach for baseline covariate adjustment for two-group comparative studies. Biometrics.

[57] Donders AR, van der Heijden GJ, Stijnen T, Moons KG (2006). Review: a gentle introduction to imputation of missing values. J Clin Epidemiol.

