# Supplementary material for: Association of pre-operative estimated GFR on post-operative pulmonary complications in laparoscopic surgeries
Source: Sci Rep. 2017 Jul 26;7:6504. doi: 10.1038/s41598-017-06842-4 (PMC5529443; doi:10.1038/s41598-017-06842-4)
Supplement: Supplementary file 1 — Supplemental Materials [file 41598_2017_6842_MOESM1_ESM.pdf]

Title:

Association of pre-operative estimated GFR on post-operative pulmonary complications in laparoscopic surgeries

Akihiro Shimomura, MD, PhD; Yoshitsugu Obi, MD, PhD*; Reza Fazl Alizadeh, MD;

Shiri Li, MD, PhD; Ninh Tuan Nguyen, MD; Michael J. Stamos, MD;

Kamyar Kalantar-Zadeh, MD, MPH, PhD; and Hirohito Ichii, MD, PhD

Supplement Figure S1. Distributions and restricted cubic splines comparing the relationship of estimated GFR with risk of post-operative pulmonary complications among 425,213 patients who underwent laparoscopic surgery (2005-2013): (A) unadjusted model, (B) case-mix adjusted model, (C) fully adjusted model. Dashed and solid lines represent OR estimates 95% CIs, respectively. Abbreviations: GFR, glomerular filtration rate.

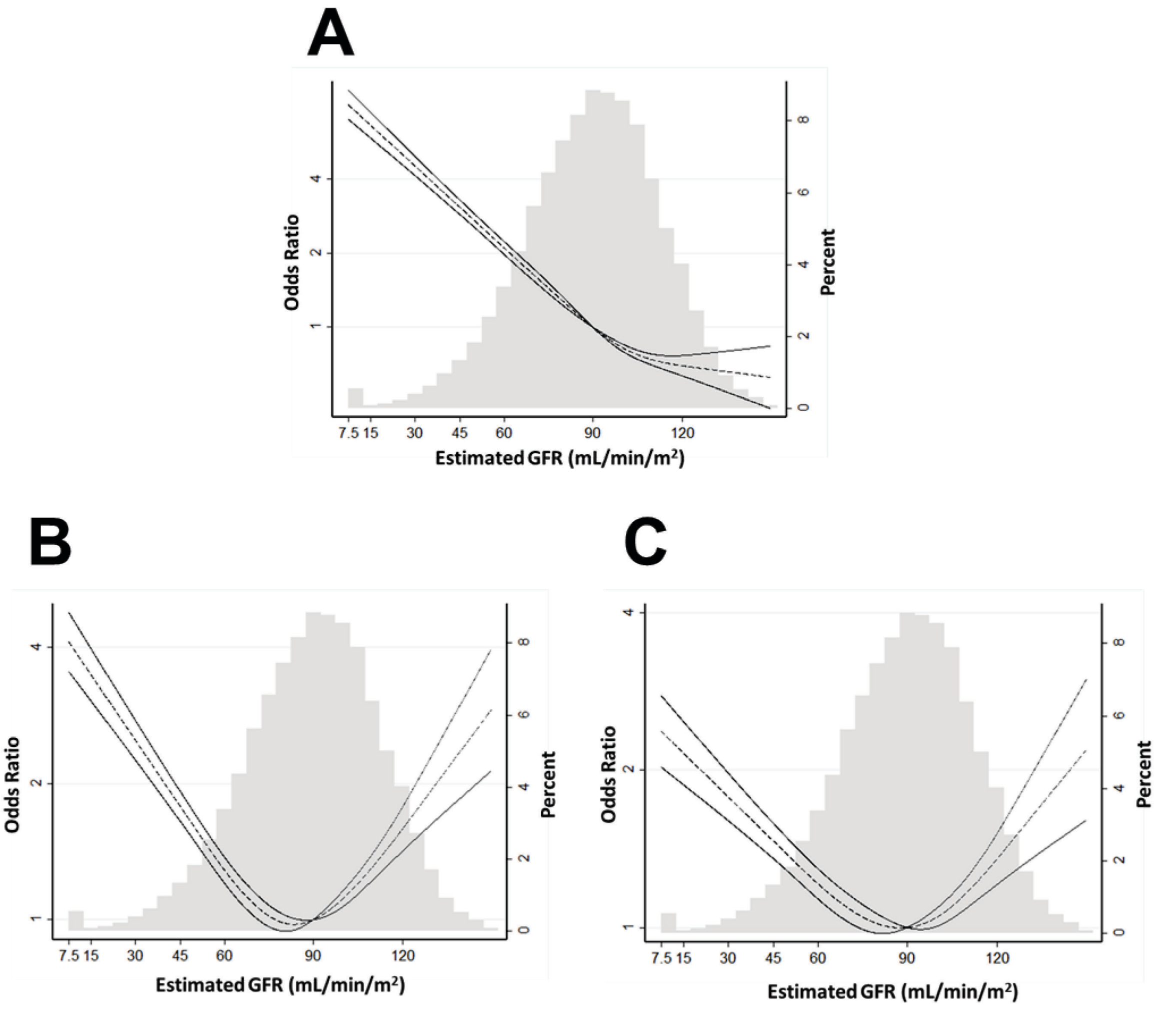

Supplemental Table S1. Association of estimated GFR with post-operative pulmonary complications with the three levels of adjustment

	Unadjusted		Case-mix adjusted		Fully adjusted	
	Odds ratio (95% CI)	*P* Value	Odds ratio (95% CI)	*P* Value	Odds ratio (95% CI)	*P* Value
Entire						
eGFR<30	6.43 (5.50 to 7.51)	<0.001	2.86 (2.43 to 3.38)	<0.001	1.83 (1.54 to 2.16)	<0.001
30≤eGFR<60	3.90 (3.56 to 4.28)	<0.001	1.53 (1.37 to 1.70)	<0.001	1.38 (1.24 to 1.54)	<0.001
60≤eGFR<90	1.60 (1.48 to 1.73)	<0.001	0.95 (0.87 to 1.03)	0.21	1.00 (0.92 to 1.09)	0.948
90≤eGFR<120	Reference		Reference		Reference	
eGFR≥120	0.74 (0.63 to 0.88)	0.001	1.45 (1.21 to 1.74)	<0.001	1.28 (1.07 to 1.53)	0.008
BMI<30						
eGFR<30	6.13 (4.88 to 7.72)	<0.001	2.27 (1.78 to 2.89)	<0.001	1.44 (1.12 to 1.85)	0.004
30≤eGFR<60	4.17 (3.65 to 4.77)	<0.001	1.37 (1.16 to 1.61)	<0.001	1.28 (1.09 to 1.51)	0.002
60≤eGFR<90	1.63 (1.44 to 1.84)	<0.001	0.83 (0.73 to 0.95)	0.008	0.92 (0.80 to 1.05)	0.224
90≤eGFR<120	Reference		Reference		Reference	
eGFR≥120	0.71 (0.54 to 0.94)	0.016	1.74 (1.29 to 2.34)	<0.001	1.33 (0.99 to 1.80)	0.058
BMI≥30						
eGFR<30	6.72 (5.43 to 8.33)	<0.001	3.47 (2.77 to 4.33)	<0.001	2.30 (1.82 to 2.90)	<0.001
30≤eGFR<60	3.65 (3.22 to 4.14)	<0.001	1.69 (1.46 to 1.96)	<0.001	1.50 (1.29 to 1.74)	<0.001
60≤eGFR<90	1.57 (1.42 to 1.75)	<0.001	1.06 (0.95 to 1.19)	0.293	1.09 (0.97 to 1.22)	0.129
90≤eGFR<120	Reference		Reference		Reference	
eGFR≥120	0.77 (0.62 to 0.95)	0.016	1.33 (1.05 to 1.67)	0.017	1.24 (0.99 to 1.57)	0.064

Abbreviations: CI, confidence interval.

Supplemental Table S2. Association of estimated GFR with post-operative non-pulmonary infectious complications with the three levels of adjustment

	Unadjusted		Case-mix adjusted		Fully adjusted	
	Odds ratio (95% CI)	*P* Value	Odds ratio (95% CI)	*P* Value	Odds ratio (95% CI)	*P* Value
eGFR<30	2.01 (1.81 to 2.24)	<0.001	1.45 (1.30 to 1.61)	<0.001	1.11 (0.99 to 1.24)	0.065
30≤eGFR<60	1.45 (1.38 to 1.53)	<0.001	0.95 (0.89 to 1.00)	0.069	0.89 (0.84 to 0.95)	<0.001
60≤eGFR<90	1.10 (1.07 to 1.14)	<0.001	0.88 (0.84 to 0.91)	<0.001	0.91 (0.87 to 0.94)	<0.001
90≤eGFR<120	Reference		Reference		Reference	
eGFR≥120	0.80 (0.75 to 0.86)	<0.001	1.12 (1.04 to 1.20)	0.002	1.04 (0.96 to 1.11)	0.33

Abbreviations: CI, confidence interval.

Supplemental Table S3. Major laparoscopic surgery Current Procedural Terminology (CPT) codes

Surgery Type	CPT codes
Cholecystectomy	47562, 47563, 47564
Gastroesophageal reflux	43279, 43280, 43283
Paraesophageal hernia	43280, 43281, 43282
Bariatric surgery	43644, 43645, 43659, 43770, 43771, 43772, 43773, 43774, 43775
Colectomy	44204, 44205, 44206, 44207, 44208, 44210, 44211, 44212
Inguinal hernia	49650, 49651
Incisional hernia	49652, 49653, 49654, 49655, 49656, 49657
Adrenalectomy	60650
Appendectomy	44970, 44979
Splenectomy	38120
Colostomy	44188, 44206, 44208
Exteriorization of intestine	44227
Ileostomy	44186, 44187
Proctectomy	45395, 45397, 45400, 45402, 45499
Nephrectomy	50543, 50545, 50546, 50548
Prostatectomy	55866
Hysterectomy	58541, 58542, 58543, 58544, 58548, 58550, 58552, 58553, 58554, 58570, 58571, 58572, 58573
Myomectomy	58545, 58546
